# Long-term care status for the elderly with different levels of physical ability: a cross-sectional survey in first-tier cities of China

**DOI:** 10.1186/s12913-023-09987-3

**Published:** 2023-09-06

**Authors:** Mingchao Zhou, Fubing Zha, Fang Liu, Jing Zhou, Xiangxiang Liu, Jiehui Li, Qingqing Yang, Zeyu Zhang, Feng Xiong, Dianrui Hou, Hongyun Weng, Yulong Wang

**Affiliations:** 1 Department of Rehabilitation, The First Affiliated Hospital of Shenzhen University, Shenzhen Second People’s Hospital, No.3002, Sungang West Road, Futian District, Shenzhen, Guangdong 518000 China; 2 Shandong University of Traditional Chinese Medicine, Jinan, Shandong China

**Keywords:** Elderly, Disability, Long term care, Activities of daily living, Longshi Scale

## Abstract

**Background:**

Long term care (LTC) services for functionally impaired senior citizens are crucial for addressing the challenges of aging. However, research on eligibility criteria and coverage of LTC in China is lacking. Our objective is to assess the current status of LTC and explore eligibility criteria and coverage for the elderly.

**Methods:**

This is a cross-sectional study conducted in two first-tier cities in China. Residents aged 65 or over were recruited from a nursing home and four primary hospitals. Participants were divided into three groups (bedridden, domestic, and community), then six grades (grade one to six) according to the Longshi Scale, and their functional ability was assessed using the Modified Barthel Index. Information such as diseases, complications, and daily care needs were collected. Nursing staff were invited to indicate patients’ needs for care. A one-way ANOVA test, Kruskal Wallis H test and Mann–Whitney U test were used to explore the differences of variables in three Longshi groups or Longshi grades.

**Results:**

Among all 1157 participants, with an average age of 80.54, 69.3% were in the bedridden group. The most common diagnosis was stroke (71.4%), with the most prevalent complication being pulmonary infection (25.2%). In the nursing assessment, basic health care, disease care, activity care, complication prevention care and psychosocial care were summarized as the five main aspects of LTC for the elderly. Feeding, bathing, drinking, bowel management and bladder management were identified as the basic care which fulfills participants’ basic physical needs in each Longshi group. Mouth care, artificial airway management, and body reposition, which can prevent immobility complications, were highly demanded by bedridden elderly.

**Conclusions:**

The elderly in grade one to three are the ones in need of LTC most. The content of LTC for elderly should include basic care which fulfills their basic physical needs and complication care which can prevent immobility complications. The evidence of this research may contribute to the design of LTC in China.

**Trial registration:**

The study design was registered in the Chinese Clinical Trial Registry (ChiCTR-2000034067, Registered 22 Jun 2020, http://www.chictr.org.cn/showproj.aspx?proj=54770).

**Supplementary Information:**

The online version contains supplementary material available at 10.1186/s12913-023-09987-3.

## Introduction

Population aging is a significant demographic trend both globally and within China, with a growing trend observed in recent years [1]. By 2050, it is estimated that the number of elderly individuals aged 65 or above in China will reach around 365 million, comprising 26.1% of the total population [2]. Currently, 40.63 million elderly people in China, accounting for 18.3% of the elderly population, face disability or semi-disability, which presents a significant challenge to the healthcare system [3]. Interventions to promote health in older age require a variety of supporting resources and systematic service arrangements from the healthcare system and the government [4]. As of the end of 2021, China has developed 358,000 elderly care institutions with 8.159 million available beds yet 45% of these beds remain unoccupied, indicating that institutional care services reach less than 10% of the elderly population [5, 6]. Most elderly individuals still rely on family-based care, but factors such as low fertility, a distorted gender ratio, and internal migration can hinder access to informal care for those from low-income families or with migrant children living far away. As a result, publicly-funded care becomes more necessary [2]. Therefore, ensuring sufficient and appropriate geriatric care for the elderly in this aging society is an urgent problem that must be addressed [7].

Long term care (LTC) aiming to provide basic assistance to elderly people with disabilities and self-care deficits in performing everyday activities, plays an important role in coping with the aging society [8]. It has been developed quickly all around the globe. European countries, such as the United Kingdom, developed acute and intermediate care systems and successfully maintained senior citizens’ physical independence in the community [9]. Asian countries, such as Japan, developed acute care and LTC settings to ensure personal care is provided to the elderly [10]. In Australia, a comprehensive LTC system was established in the community—whether in terms of residential care, hospitals, or other types of institutional care—to meet the various personal needs of the elderly [11]. Among these countries, the primary commonality in LTC for the elderly is the need for LTC needs assessment and functional disability level determination. However, the standards for these assessments vary across different regions [12].

Facing a significant increase of LTC demands, the Chinese government plans to establish a LTC system that is primarily community and homecare-based, and supplemented by public and private nursing homes [1]. To this end, China implemented a national long-term care insurance (LTCI) plan across 15 pilot cities since 2016 [13]. The LTCI can cover approximately 85–90% of LTC service costs for the elderly who are aged 60 or over, and are covered by the national public health insurance scheme. A study by Fudan University found that the introduction of LTCI in China has reduced the length of hospital stay, inpatient expenditures, and health insurance expenditures in tertiary hospitals by 41.0%, 17.7%, and 11.4% respectively during 2016–2017 [14]. Therefore, LTC facilities have developed rapidly since 2016. At the same time, there is an increasing trend among elderly people with limited physical capacity choosing LTC institutions as their ideal retirement destination. This can not only help the elder generation reduce financial burden on their family but also aids the government in distributing public medical resources within the national healthcare system appropriately [15].

However, the LTC system in China is negatively affected by the growing residential (institutional) care sector, slow development of home and community-based services, shortage of LTC workers, weak quality regulations, and insufficient organized funding [16, 17]. Before meaningful progress can be made to prepare the country for its rapid entrance into an ageing society, several challenges need to be understood and addressed. First, care needs must be assessed comprehensively; second, service capacity must to be improved; third, nursing resources need to be allocated properly [1]. In order to deal with these challenges, it is necessary to learn about which diagnosis and complications LTC should mainly focus on, identify the population eligible for LTC most, and understand what LTC for the elderly should comprise. Thus, in this study, we investigate the current application of LTC in hospitals and nursing homes in first-tier cities of China where LTC has been successfully integrated into the national healthcare system. The purpose is to understand the main diagnoses and complications of LTC subjects, as well as identify the suitable population eligible for LTC and explore the care needs of elderly with different levels of physical ability. This can provide evidence for the development of specialized LTC standards.

## Material and methods

### Design

This is a cross-sectional study conducted by the rehabilitation department of Shenzhen Second People’s Hospital, Shenzhen, China. It is a sub-study of a clinical research “The implementation of Longshi Scale” which explored the application value of the Longshi Scale nation-wide [18].

### Study population

Participants were screened and recruited from a nursing home in Shanghai and four primary hospitals in Shenzhen where LTC services had been comprehensively developed and incorporated into the healthcare system and the public welfare system were identified. Shanghai and Shenzhen were pilot cities that implemented the LTCI plan in China and launched the plan in 2017 and 2020, respectively. Unlike tertiary and secondary hospitals which only provide short-term inpatient services, primary hospitals and nursing homes can provide long-term inpatient care for disabled elderly.

In our study, the nursing home in Shanghai was selected not only because it is one of the largest nursing homes in Shanghai and admits elderly residents with a large variety of functional ability levels but also because it had already implemented LTCI. The selection of the primary hospitals in Shenzhen followed the same principle. Among all the primary hospitals in Shenzhen, the ones providing LTC and had implemented LTCI were included in our study.

**Fig. 1:**
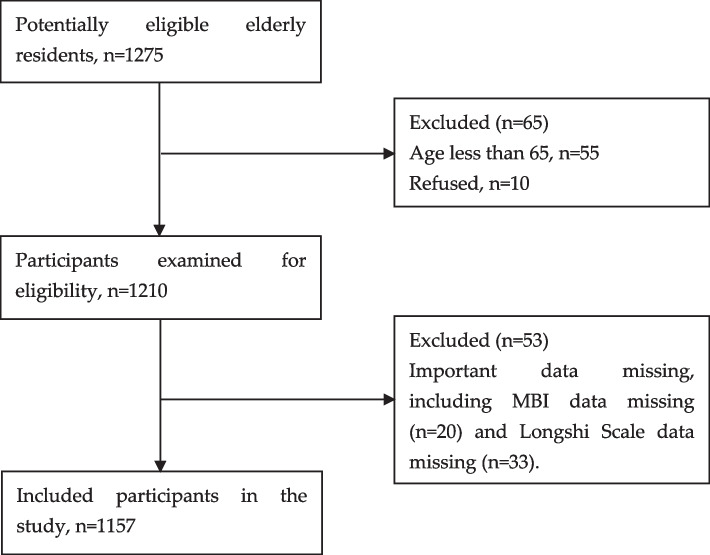
Study flowchart

The inclusion criteria were 1) residents who were aged 65 or over, 2) residents who received long-term care in hospitals or nursing home facilities, 3) participants who signed informed consent forms. The exclusion criteria were 1) residents with mental illnesses, 2) residents who were unwilling to cooperate with the study. A total of 1,275 participants were initially screened for eligibility, with 1,210 of them confirmed as eligible. After excluding individuals with important missing data (including MBI data missing (*n* = 20) and Longshi Scale data missing (*n* = 33)), a final sample of 1,157 elderly residents was recruited for this study. Figure 1 shows the flowchart of the study.

### Instruments

All the data were collected using questionnaires. The contents of the questionnaire were as follows: (1) Demographic information, including sex, age, weight and height; (2) Disease information, including primary diagnoses, complications and comorbidities; (3) Functional assessment information, including assessment of Modified Barthel Index and Longshi Scale; (4) Care needs information, including nursing assessment and nursing time used.

#### Longshi Scale

Longshi Scale is an innovative functional assessment tool developed by Yulong Wang, the head of our rehabilitation institution. It is a pictorial scale and evaluates activity of daily living (ADL) based on patients’ daily activity range and level of dependency [18]. First, participants were divided into three groups based on their daily activity range: bedridden, domestic, and community. The bedridden group could not get out of bed independently, the domestic group could get out of bed but not go outdoors independently, and the community group could go outdoors independently. Then, different sets of assessment items were used to calculate the Longshi score in each group, ranging from three to nine points. Within each group, participants were further divided into two grades based on their Longshi scores. Ultimately, all participants were divided into six grades. Supplementary Fig. 1 shows the details of the Longshi scale classification system.

The Longshi Scale was made the national standard of ADL measurement of China in 2019 (No. GB/T 37103–2018) [19]. Since then, it has been widely used among first-tier cities all over China. We have proven that the Longshi Scale was reliable and valid to assess the ADL of functionally disabled patients [18] and that this scale was highly correlated with the Modified Barthel Index (MBI) in ADL assessment [20]. As it is a newly designed functional assessment scale, all registered healthcare workers completed the training sessions for Longshi Scale before participants’ evaluation.

#### MBI

To investigate the basic physical activities, participants’ functional ability was evaluated using MBI. The MBI was adapted by Shah et al [21] in 1989 based on the Barthel Index (BI), and its Chinese version was published by Leung and Shah et al. in 2007 [22]. It has been reported that the functional division of MBI is more subtle than BI, [23] and MBI has better test–retest reliability and relatively lower random measurement error than the original BI [24]. In current practice, MBI or BI is one of the most commonly used ADL assessment tools to evaluate patient’s physical and functional ability in China [25]. Thus, this scale was used in the LTC facilities where we conducted our study.

The MBI score ranges from 0 (complete dependence of ADL) to 100 points (complete independence of ADL). There are ten assessing items in this scale, including bowel management, bladder management, feeding, bathing, grooming, clothing, toilet use, transfer, walking, and climbing stairs [23]. These ten items were used as a guide to identify the area of patients’ needs. Each MBI item has five levels, ranging from level one (complete dependence of activity) to level five (complete independence of activity). Except for participants in level five, who were determined as independent ones, all remaining participants required some level of assistance or supervision to perform daily activities and were identified as dependent. In each MBI assessing item, the ratio of dependent patients was calculated to explore which ADL participants required assistance most. The evaluation process of the MBI was based on previous literature reports [22].

#### Nursing assessment

What should be included in LTC for the elderly with functional disability needs to be carefully designed using evidence. In addition to the investigation on participants’ functional disabilities, we also explored their needs from the nursing perspective. In this study, nursing staff were interviewed face-to-face using questionnaires to indicate specific nursing care provided to each participant. Fourteen themes of nursing care required by participants in the day time from waking up in the morning until settling in bed at night were listed. Within these fourteen nursing care themes, six items (bathing, feeding, bowel management, bladder management, grooming, transfer) were consistent with the MBI items while the remaining eight items (drinking, hypertension management, blood glucose management, assistive motion exercise, mouth care, body reposition, artificial airway management, entertainment) were not included in MBI assessment. All these fourteen items were summarized as five aspects, including basic health care, disease care, activity care, complication prevention care, and psychosocial care. According to Maslow's hierarchy of needs theory, bathing, feeding, drinking, grooming, and bowel and bladder management are categorized as basic health care as they aim to fulfill fundamental physiological needs. Blood pressure management and blood glucose management are related to disease care, addressing specific medical conditions. Assisting patients with assistive motion exercise and transfers falls under activity care as they aim to increase patients' physical mobility and prevent prolonged immobility. Mouth care and artificial airway management contribute to oral and respiratory hygiene, preventing lung infections, while proper body positioning helps prevent complications such as deep vein thrombosis and pressure ulcers caused by prolonged bed rest, categorized as complication prevention care. Entertainment addresses patients' psychological needs, often overlooked but crucial for their mental well-being, and thus categorized as psychosocial care.

The care needs ratio is calculated by dividing the number of individuals requiring nursing care by the total number of individuals. The nursing care time is calculated by multiplying the duration of each care theme by the number of times a day that care was provided.

### Data collection

All the data for this study were collected between July 2021 to August 2021. To ensure data collection consistency, prior to conducting this study, we recruited a total of 34 allied health practitioners (including physiotherapists, occupational therapists and speech pathologists) from the nursing home and four primary healthcare hospitals. They received unified training on the research procedures, clinical data collection and recording methods, as well as the assessment methods of the MBI and Longshi scale. Additionally, we recruited 33 nursing staff members (including registered nurses and nursing assistances) and provided them with unified training on the criteria for collecting information regarding personal needs and care required. Prior to data collection, the investigator explained the study procedure and protocol in detail and obtained informed consent from each nursing participant, elderly participant, and the guardian of disoriented participants who suffered from dementia or consciousness disorder. Considering the labor cost, this study only investigated care needs during the day, from residents waking up in the morning until settling in bed at night. All data were recorded with an electronic questionnaire system on smart phones and saved on the website (https://www.mikecrm.com). Data deletion, modification or sharing without permission were prohibited. During the data collection process, the database was supervised by a data administrator Fubing Zha, who conducted regular reviews to ensure data integrity and accuracy. Once unreasonable data were noticed, the data administrator would check with the assessor and correct any errors according to the actual situation. For example, if a patient is assessed with an MBI score of 100 but is categorized as bedridden according to the Longshi scale, or if the MBI score is 0 but the Longshi scale categorizes the patient as a community-member, it is considered as inconsistent data. In such cases, the data administrator will collaborate with the assessors to verify and correct the data.

### Data analysis

All analyses were performed using SPSS 21.0 (IBM, New York, USA). Mean ± standard deviation or median and interquartile was used to describe continuous variables. Individuals with important data missing were excluded from the analysis. A one-way ANOVA test was used to explore the differences of age and BMI across the three Longshi groups. The Kruskal Wallis H test was performed to explore the differences of categorical data across the three Longshi groups. The Mann–Whitney U test was used to analyze the differences of nursing care needs among different Longshi groups or Longshi grades. Pearson and Spearman correlation analyses were used to test the association between MBI and clinical characteristics. A *P*-value less than 0.05 was considered as statistically significant.

## Results

Clinical characteristics of the elderly residents across the three Longshi groups

The majority of participants (69.3%) were in the bedridden group, while only 5.4% belonged to the community group. The average age of participants in each Longshi group was around 80 years, and there was no statistically significant differences among the groups (80.37 ± 8.18, 81.14 ± 8.19, 79.94 ± 7.68, *p* = 0.267). The duration of disease ranged from 1 month to 3 years. The most common diseases among participants in all three groups were stroke (72.3%, 70.6%, 62.9%, *p* = 0.272) and hypertension (66.7%, 67.9%, 53.2%, *p* = 0.076), followed by heart disease (30.3%, 32.1%, 25.8%, *p* = 0.605), diabetes (32.5%, 35.5%, 27.4%, *p* = 0.412), and dementia (8.9%, 6.5%, 3.2%, *p* = 0.162). The incidence of complications in bedridden patients, including pulmonary infections (32.0%, 11.3%, 1.6%, *p* < 0.001), urinary tract infections (9.9%,, 6.5%, 1.6%, *p* = 0.028), and deep vein thrombosis (16.0%, 9.6%, 3.2%, *p* = 0.001), were significantly higher than that in domestic and community individuals. Besides, the incidence of pulmonary infection (*r* = -0.357, *p* < 0.001), deep vein thrombosis (*r* = -0.153, *p* < 0.001), urinary tract infection (*r* = -0.087, *p* = 0.003) and pressure ulcers (r = -0.081, *p* = 0.006) all revealed a significant negative association with MBI scores. The characteristics of the participants are presented in Table 1.

**Table 1:** Clinical characteristics of elderly participants in three Longshi groups

Characteristics	Longshi Scale					
	Overall (*n* = 1157)	Bedridden (*n* = 802, 69.3%)	Domestic (*n* = 293, 25.3%)	Community (*n* = 62, 5.4%)	F/H	*P* value
Sex						
Male, n, %	567 (49.0)	390 (48.6)	145 (49.5)	32 (51.6)	0.241	0.886
Female, n, %	590 (51.0)	412 (51.4)	148 (50.5)	30 (48.4)		
Age (years, Mean, SD)	80.54 ± 8.16	80.37 ± 8.18	81.14 ± 8.19	79.94 ± 7.68	1.154	0.316
Duration of disease (months, Median, IQR)	10.63(1.58–36.92)	10.77(1.97–35.07)	11.63(1.07–48.70)	3.47 (0.33–36.53)	3.729	0.155
BMI (kg/m^2^, M, SD)	21.58 ± 3.50	21.35 ± 3.58	22.08 ± 3.26	22.20 ± 3.23	5.393	0.005
Living habit						
History of smoke (n, %)	49 (4.2)	33 (4.1)	12 (4.1)	4 (6.5)	0.783	0.676
No history of smoke (n, %)	1108 (95.8)	769 (95.9)	281 (95.9)	58 (93.5)		
History of drinking (n, %)	32 (2.8)	18 (2.3)	11 (3.8)	3 (4.8)	2.870	0.238
No history of drinking (n, %)	1125 (97.2)	784 (97.8)	282 (96.2)	59 (95.2)		
Disease						
Stroke (n, %)	826 (71.4)	580 (72.3)	207 (70.6)	39 (62.9)	2.602	0.272
Hypertension (n, %)	767 (66.3)	535 (66.7)	199 (67.9)	33 (53.2)	5.141	0.076
Heart disease (n, %)	353 (30.5)	243 (30.3)	94 (32.1)	16 (25.8)	1.004	0.605
Diabetes (n, %)	382 (33.0)	261 (32.5)	104 (35.5)	17 (27.4)	1.772	0.412
Dementia (n, %)	92 (8.0)	71 (8.9)	19 (6.5)	2 (3.2)	3.640	0.162
Chronic obstructive pulmonary disease (COPD) (n, %)	41 (3.5)	30 (3.7)	9 (3.1)	2 (3.2)	0.300	0.861
Cancer (n, %)	18 (1.6)	14 (1.7)	3 (1.0)	1 (1.6)	0.731	0.694
Complications						
Pulmonary infection (n, %)**	291 (25.2)	257 (32.0)	33 (11.3)	1 (1.6)	68.455	< 0.001
Urinary tract infection (n, %)*	99 (8.6)	79 (9.9)	19 (6.5)	1 (1.6)	7.138	0.028
Deep vein thrombosis (n, %)**	158 (13.7)	128 (16.0)	28 (9.6)	2 (3.2)	13.496	0.001
Pressure ulcers (n, %)*	58 (5.0)	47 (5.9)	10 (3.4)	1 (1.6)	4.286	0.117

*MBI* Modified Barthel Index, *BMI* Body Mass Index, *SD* Standard deviation, *IQR* Interquartile range, *P* *p* value among bedridden group, domestic group and community group, One-way ANOVA test for age and BMI, Kruskal Wallis H test for other characteristics. F: F value for One-way ANOVA test. H: H value for Kruskal Wallis H test. *Significant association between MBI and complications, spearman correlation analysis, *p* < 0.05, **: *p* < 0.001

Functional disabilities of elderly residents across the three *Longship group.*

In our study, the number of participants in grade five and six was small (only 4 in grade five and 58 in grade six). Since both grade five and six belonged to the community group, the participants from these two grades were combined as the community group for statistical analysis.

The average MBI scores of participants in grades one, two, and three were significantly lower than those of the community group (5.79 ± 11.75, 34.83 ± 20.52, 51.97 ± 18.90 vs 68.95 ± 14.14, *p* < 0.001). However, the average MBI score of participants in grade four was similar to that of the community group (67.13 ± 15.40 vs 68.95 ± 14.14, *p* = 0.427). More specifically, the average MBI scores of participants in grades one, two, and three were significantly lower than those of the community group in each MBI assessing item (*p* < 0.001), except for grade three participants' average MBI score in the transfer item. On the other hand, the MBI score of participants in grade four showed no significant difference compared to the community group participants in all MBI items, except for climbing stairs (*P* > 0.05). Table 2 provides further details regarding the same.

**Table 2:**
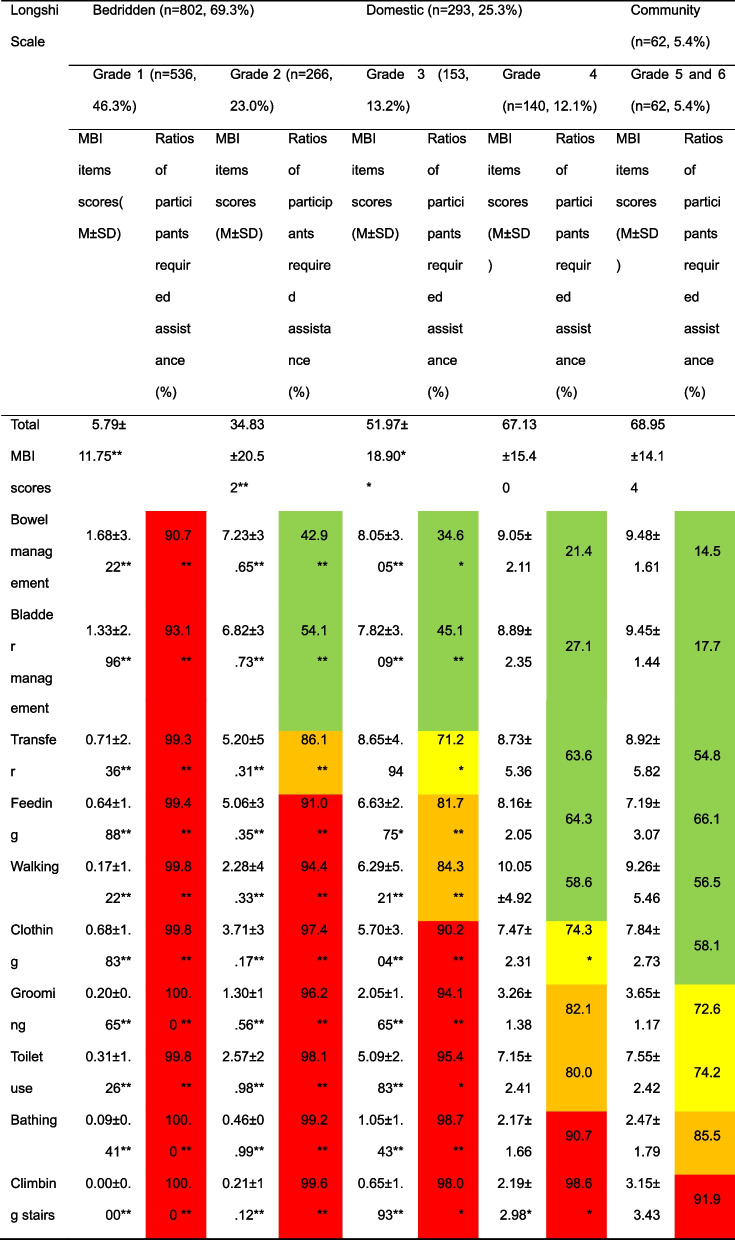
ADL disabilities of elderly participants in three Longshi groups

^*^Compared with Community group (grade 5 and 6), *P* < 0.05; ** compared with Community group (grade 5 and 6), *P* < 0.001. Mann–Whitney U test. Red: over 90%, orange: 80%-90%, yellow: 70%-80%, green: lower than 70%

From the ten MBI assessing items, climbing stairs (99.0%), bathing (97.8%), toilet use (95.1%), grooming (94.7%), and clothing (92.7%) were the top five activities in which participants required assistance. Of these, grade one participants required assistance in all ten items, with a majority (more than 90%) needing assistance. In comparison, grade two residents required assistance in seven out of ten items, grade three residents in five out of ten items, grade four residents in two out of ten items, and community group participants in only one item (climbing stairs). The proportions of participants in grades one, two, and three who required assistance in all MBI activities were significantly higher than those in the community group (*p* < 0.05), while the proportions of grade four participants who required assistance were only higher in clothing and climbing stairs compared to the community group (*p* < 0.05). Table 2 presents the percentage of residents requiring assistance in each MBI item, with different color highlights representing different percentage ranges to compare the care needs among residents with varying functional abilities.

Nursing care required by elderly across the three *Longshi groups*.

Among the fourteen nursing care themes required by participants in the three Longshi groups, the top six common needs were bathing (92.6%, 84.1%, 75.0% in males; 91.5%, 85.1%, 83.3% in females), feeding (91.0%, 80.0%, 87.5% in males; 91.0%, 82.4%, 86.7% in females), drinking (87.9%, 79.3%, 78.1% in males; 89.3%, 78.4%, 90.0% in females), bowel management (87.4%, 80.0%, 68.8% in males; 86.2%, 76.4%, 90.0% in females), bladder management (86.9%, 77.2%, 78.8% in males; 85.0%, 77.0%, 90.0% in females), and grooming (79.2%, 78.6%, 68.8% in males; 70.6%, 71.6%, 76.7% in females). Bedridden residents had a significantly higher need for assistance in mouth care (68.2% vs 26.9%, 25.0% in males; 64.6% vs 29.1%, 20.0% in females), body repositioning (68.5% vs 16.6%, 6.3% in males; 60.0% vs 14.2%, 3.3% in females), and artificial airway management (40.5% vs 2.8%, 0.0% in males; 25.2% vs 1.4%, 0.0% in females) compared to participants in the domestic and community groups. However, their demand for assistance in entertainment (36.4% vs 63.4% in males; 30.6% vs 62.2% in females) and transfer (38.2% vs 51.7% in males; 25.5% vs 38.5% in females) was significantly lower than participants in the domestic group. In all nursing care themes, except for assistive motion exercise and entertainment, the needs of domestic residents and community residents showed no significant difference (*P* > 0.05).

Among all nursing care themes, feeding requires the longest nursing time in the three Longshi groups, with a median of 60 min for both males and females. Additionally, bathing (median 30 min for both males and females in the three Longshi groups) and drinking (median 25, 23, 20 min in males; median 25, 19, 15 min in females in the three Longshi groups) also requires a significant amount of nursing time. On the other hand, entertainment is relatively less required, but it still takes considerable time to perform (median 40, 45, 60 min in males; median 40, 55, 60 min in females in the three Longshi groups).

When comparing males and females, it was found that bedridden male residents had significantly higher needs for assistance in grooming (79.2% vs 70.6%), assistive motion exercise (69.7% vs 57.0%), body repositioning (68.5% vs 60.0%), transfer (38.2% vs 25.5%), and artificial airway management (40.5% vs 25.2%) compared to bedridden female participants. Additionally, bedridden male residents required significantly more time for assistance in feeding, bowel management, and mouth care than their female counterparts. However, there were almost no significant differences in care needs and care time between males and females in domestic and community residents (*p* > 0.05). More details regarding the same are shown in Table 3.

**Table 3:** Care needs of elderly participants in practical nursing programs

Nursing aspects	Nursing items	Male						Female					
		Bedridden (*n* = 390, 68.8%)		Domestic (*n* = 145, 25.6%)		Community (*n* = 32, 5.6%)		Bedridden (*n* = 412, 69.8%)		Domestic (*n* = 148, 25.1%)		Community (*n* = 30, 5.1%)	
		Care needs (N, %)	Care time (min, Median, quartiles)	Care needs (N, %)	Care time (min, Median, quartiles)	Care needs (N, %)	Care time (min, Median, quartiles)	Care needs (N, %)	Care time (min, Median, quartiles)	Care needs (N, %)	Care time (min, Median, quartiles)	Care needs (N, %)	Care time (min, Median, quartiles)
Basic health care	bathing	361, 92.6%*#	30, 20–40*#	122, 84.1%	30, 20–35	24, 75.0%	30, 20–30	377, 91.5%*	30, 20–40	126, 85.1%	30, 20–36	25, 83.3%	30, 20–30
	feeding	355, 91.0%**	60, 50–90&	116, 80.0%	60, 52–89	28, 87.5%	60, 45–90	375, 91.0%*	60, 45–90	122, 82.4%	60, 45–90	26, 86.7%	60, 50–90
	drinking	343, 87.9%*	25, 15–40	115, 79.3%	23, 15–38	25, 78.1%	20, 12–30	368, 89.3%*	25, 15–40#	116, 78.4%	19, 15–30	27, 90.0%	15, 12–30
	bowel management	341, 87.4%*#	15, 10–20*#&	116, 80.0%	11, 10–15	22, 68.8%&	10, 5–15	355, 86.2%*	10, 9–20	113, 76.4%	10, 8–15	27, 90.0%	10, 10–10
	bladder management	339, 86.9%*#	20, 12–30*	112, 77.2%	15, 10–30	22, 68.8%	17, 12–24	350, 85.0%*	18, 12–30	114, 77.0%	15, 12–30	27, 90.0%	16, 12–25
	grooming	309, 79.2%&	20, 10–30	114, 78.6%	20, 10–30	22, 68.8%	15, 10–30	291, 70.6%	20, 10–30	106, 71.6%	20, 10–30	23, 76.7%	20, 10–20
Disease care	Hypertension management	334, 85.6%**&	5, 3–10*#&	102, 70.3%	5, 3–6	25, 78.1%	3, 2–5	331, 80.3%	5, 3–6*	117, 79.1%	4, 3–5	23, 76.7%	3, 2–5
	Blood glucose management	156, 40.0%*&	4, 2–9	42, 29.0%	3, 2–8	11, 34.4%	4, 3–6	120, 29.1%	4, 2–8	50, 33.8%	4, 2–6	10,33.3%	4, 2–5
Activity care	assistive motion exercise	272, 69.7%*##&&	30, 30–40	84, 57.9%#	30, 30–52	12, 37.5%	40, 25–60	235, 57.0%	30, 20–45	89, 60.1%	30, 20–50	17, 56.7%	40, 20–90
	transfer	149, 38.2%*&&	25, 10–40*	75, 51.7%&	30, 20–60	12, 37.5%	28, 20–50	105, 25.5%*	20, 10–40	57, 38.5%	30, 20–60	7, 23.3%	30, 30–60
Complication prevention care	mouth care	266, 68.2%**##	10, 5–15&	39, 26.9%	10, 10–20&	8, 25.0%	10, 6–15	266, 64.6%**##	10, 5–11	43, 29.1%	10, 5–11	6, 20.0%	10, 10–15
	body reposition	267, 68.5%**##&	30, 15–60	24, 16.6%	30, 18–40&	2, 6.3%	20, 10–30	247, 60.0%**##	30, 15–60	21, 14.2%	12, 10–24	1, 3.3%	24, 24–24
	artificial airway management	158, 40.5%**##&&	20, 10–30	4, 2.8%	16, 9–20	0, 0.0%	-	104, 25.2%**##	17, 10–30	2, 1.4%	20, 15–25	0, 0.0%	-
Psychosocial care	entertainment	142, 36.4%**	40, 30–60	92, 63.4%#	45, 30–60	14, 43.8%&	60, 30–60	126, 30.6%**##	40, 30–60	92, 62.2%	55, 30–60	22, 73.3%	40, 30–60

^*^Compared with domestic group, *p* < 0.05; **: Compared with domestic group, *p* < 0.001; #: Compared with community group, *p* < 0.05; ##: Compared with community group, *p* < 0.001; &: Compared between male and female, *p* < 0.05; &&: Compared between male and female, *p* < 0.001; Mann–Whitney U test

## Discussion

In this study, the questionnaires distributed to elderly participants, health practitioners and nursing staff of the LTC facilities revealed the common diseases and complications suffered by the elderly. In addition, we found that the combined use of MBI and Longshi Scale can accurately identify the elderly population eligible for LTC, which may help decision makers gather information needed for optimal resource allocation. The component of LTC should be designed through the combination of basic physical and essential individual needs to ensure that all needs are met.

### The clinical characteristics of the elderly in LTC

Stroke is the most prevalent cerebrovascular disease and the leading cause of death and disability in China and worldwide. A study conducted in the Netherlands found that cerebrovascular disease was the strongest predictor of nursing home admission, with a relative risk ratio (RRR) of 11.5 [26]. Guccione AA demonstrated that compared to heart disease and diabetes, stroke was significantly associated with functional limitations [27]. Consistent with the results of a previous study, [28] our study also found that stroke is the most common chronic disease among elderly residents, followed by hypertension, heart disease, and diabetes. Hypertension and diabetes, which are considered important risk factors for stroke, can contribute to the development of cerebrovascular disease [29, 30]. Therefore, our findings suggest that cerebrovascular diseases should be one of the primary diseases covered by LTC. Accordingly, LTC facilities need to develop specialized care programs based on the functional characteristics of stroke patients. Health policies should prioritize primary care, such as regular screening and control of blood pressure and blood glucose, in order to delay disease onset and reduce LTC cost [31].

Pulmonary infection is the top complication exhibited in all elderly residents, especially in bedridden members. We also noticed that the incidence of pulmonary infection in our study was much higher (25.2% vs 3.39%) than that of previous study [32] where its participants were bedridden patients in tertiary and secondary hospitals. One plausible explanation is that prolonged bed rest with little mobilization in LTC facilities can lead to repeated lung infections. It has been reported that pulmonary infection is the independent risk factors for death in hospitalized bedridden patients [33]. Pneumonia could cause a chronic inflammatory response which could increase cardiovascular mortality risk [34]. Therefore, in our study, we noticed that the respiratory care such as mouth care, artificial airway management and body repositioning are widely utilized among bedridden elderly individuals, with the main aim of eliminating mucus, improve breathing and preventing pulmonary infections associated with long-term bed rest.

Besides, consistent with a previous study, [35] we found that urinary tract infection, deep vein thrombosis and pressure ulcers are prevalent among bedridden participants. All these conditions are considered significant complications resulting from immobility, [36] and research has shown that they are associated with prolonged hospital stays and increased mortality rates [37]. Studies have also indicated that physical exercise can provide benefits for hospitalized patients, such as reducing the occurrence of deep vein thrombosis and urinary tract infections, as well as lowering the incidence of pneumonia [38].

Therefore, we can conclude that preventative care should be included in LTC and should be an essential part of it. Mouth care and artificial airway management can help prevent pneumonia while body reposition, assistive motion exercise, transfer out of bed and taking a walk can prevent deep vein thrombosis, pressure ulcers and urinary tract infection. It is suggested that these practices should be integrated in nursing care plan to prevent immobility complications for elderly in LTC facilities.

### The eligibility for LTC among the elderly

In our study, the combination of the MBI and Longshi Scale was used to identify the population requiring LTC. We observed that the majority of elderly residents (69.3%) in the study were classified as bedridden, while only 5.4% belonged to the community group. Additionally, the average MBI score of bedridden residents was significantly lower than that of community residents, and the proportion of bedridden residents requiring assistance with the ten MBI activities was significantly higher compared to the community residents. Based on these findings, it can be suggested that the population requiring LTC the most consists of bedridden elderly individuals who heavily rely on others to perform ADL. Conversely, senior citizens classified in the community group may not require LTC at all.

Identifying the needs for LTC among the elderly in the domestic group can be challenging due to variations in their self-care abilities and activity restrictions. To address this, we applied the Longshi Scale to divide the participants in the domestic group into grade three and grade four. Compared to the community group, grade three elderly individuals demonstrated lower functional ability and independence in ADL, whereas grade four residents exhibited similar ADL functional ability and level of independence as the community residents. Furthermore, the average MBI score of grade three residents (51.97) was significantly lower than that of grade four residents (67.13). According to the disability assessment standard for LTC (trial) issued by the Chinese government in July 2021, participants with a BI score below 60 are eligible to apply for designated nursing service institutions for long-term care [39]. Previous studies have mostly reported MBI or BI scores ranging from 10 to 49 for residents in LTC facilities [40]. Based on these findings, it can be concluded that grade three elderly individuals have a greater need for LTC, while grade four individuals, similar to those in the community group, may not necessarily require LTC.

Consequently, we suggest that the elderly in Longshi grades one, two, and three are eligible for LTC. This finding can assist the government and decision-makers in establishing more precise admission criteria for LTC. In fact, Shenzhen has already implemented the Longshi Scale as the criterion for classifying LTC levels since the beginning of 2022 [41].

### The coverage content of LTC for the elderly

A well-established LTC plan is essential for elderly individuals with limited physical or functional abilities to maintain a basic standard of living. However, the current LTC practice in China lacks standardization and specialization [42]. Therefore, in this study, we tried to explore the indispensable components that should be included in LTC by investigating participants' functional disabilities.

As recommended in previous studies, the quality indicators for LTC facilities include hypertension, vision and hearing difficulties, oral health problems, mobility problems, washing, dressing and brushing teeth difficulties, urinary incontinence, feelings of loneliness and lack of autonomy [43]. These indicators can be categorized into five aspects, including basic health care (care for vision and hearing difficulties, dressing and brushing teeth difficulties), disease care (care for hypertension and urinary incontinence), activity care (care for mobility problems), complication prevention care (care for oral health problems) and psychosocial care (care for feeling of loneliness and lack of autonomy). These findings align well with our study. We consulted nurses and nursing assistants who provided valuable insights from a nursing perspective. Based on their assessments, we found that the fourteen nursing items could also be summarized into the aforementioned five aspects, emphasizing the importance of these aspects in LTC for the elderly [44]. Additionally, our study identified the top five common nursing care needs across all functional levels, which include feeding, bathing, drinking, bowel management, and bladder management. These needs are fundamental for daily living and require significant nursing time. Moreover, they are particularly vulnerable to deterioration among the elderly, [45] emphasizing the need to prioritize these aspects in LTC facilities.

Results from functional assessment and nursing assessment also revealed that participants with varying levels of disability have distinct personal care needs. Particularly for bedridden participants, there is a greater need for assistance in complication prevention care (mouth care, artificial airway management, body reposition) as these nursing interventions help prevent immobility-related complications like aspiration pneumonia and pressure ulcers [46]. Evidence suggests that improved oral care can reduce the risk of developing aspiration pneumonia in the elderly [47]. Additionally, a review has demonstrated the effectiveness of a pressure ulcer prevention program in LTC facilities, with timely body repositioning playing a pivotal role [12]. On the other hand, bedridden participants require less assistance in entertainment activities compared to those in the domestic and community groups, indicating the importance of addressing the psychosocial needs of individuals with better functional abilities. These findings highlight the need for personalized treatment based on patients' motor abilities and the necessity of tailoring LTC content to different levels of independence to meet individual needs and avoid resource wastage.

According to the results of our study, we recommend that LTC should include basic care, which encompasses feeding, bathing, drinking, and bladder and bowel management, thereby fulfilling the basic physical needs of elderly residents. In addition, complication prevention care, such as mouth care, artificial airway management, and body repositioning, should be provided to prevent immobility-related complications, particularly for bedridden elderly individuals. Furthermore, it is important not to overlook psychosocial care for non-bedridden elderly individuals.

### Limitations

There are several limitations to this study. Firstly, it is important to note that this was an observational study conducted with elderly participants recruited from a nursing home and primary hospitals in Shanghai and Shenzhen. Therefore, the generalizability of the study results may be limited. Additionally, the study focused on LTC in first-tier cities due to the prevalence of the Longshi Scale and the more developed LTC facilities in these areas. To enhance the applicability of the findings, future research should include a multi-centered approach and encompass other cities in China. Furthermore, the nursing care themes in the questionnaire were determined based on nursing assessments and care plans from primary hospitals and nursing homes. However, it is possible that some aspects of nursing care were not fully captured, leading to potential gaps in the nursing details. Another limitation is that the study only investigated the care needs during daytime (from morning to night). As a result, the findings may not fully reflect the nursing requirements during nighttime. It is essential to consider this aspect in future research. Lastly, this study did not examine the care needs of the elderly population receiving care in the community or at home. Therefore, a comparison of the acceptance and effectiveness of different modes of LTC among the elderly could not be conducted. This aspect should be explored in future studies to provide a more comprehensive understanding of their needs.

## Conclusions

Our study revealed that cerebrovascular disease and immobility-related complications, particularly pulmonary infections, were prevalent among the elderly requiring LTC. This highlights the importance of specialized nursing training for common diseases and the implementation of effective preventive measures for common complications in LTC for the elderly. The elderly classified in Longshi grades one, two, and three were the most in need of LTC and eligible for it. LTC should encompass basic health care, disease care, activity care, complication prevention care and psychosocial care. Basic care, including feeding, bathing, drinking, and bladder and bowel management, fulfills the most fundamental living needs of the elderly. Additionally, essential care, such as mouth care, artificial airway management, body reposition, can help prevent major complications in immobile elderly individuals. This article specified who is eligible and what should be covered in LTC in China, and the evidence of this article may contribute to the design of LTC in China’s health policy.

### Supplementary Information

**Additional file 1.**

## Data Availability

The datasets generated and analyzed during the current study are included in this published article, as well as available from the corresponding author (Yulong Wang or first author (Mingchao Zhou) on reasonable request.

## Abbreviations

LTC: Long time care
LTCI: Long-term care insurance
BI: Barthel Index
MBI: Modified Barthel Index
ADL: Activities of daily living
