# Supplementary figures and images for: Long-term care status for the elderly with different levels of physical ability: a cross-sectional survey in first-tier cities of China
Source: BMC Health Serv Res. 2023 Sep 6;23:953. doi: 10.1186/s12913-023-09987-3 (PMC10481569; doi:10.1186/s12913-023-09987-3)

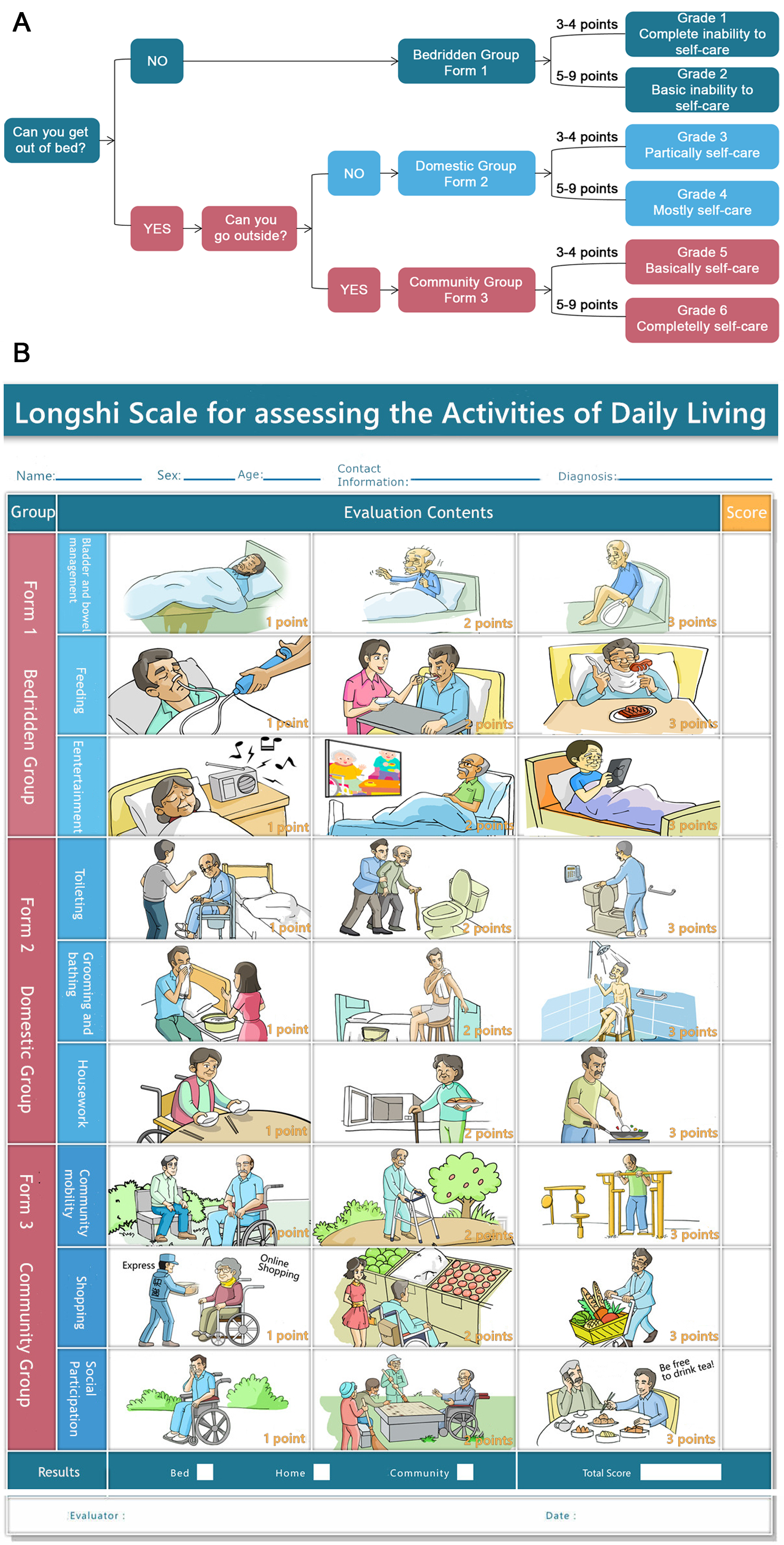

Supplement: Supplementary file 1 — Additional file 1. [file 12913_2023_9987_MOESM1_ESM.tif]
